# A Putative Effector LtCSEP1 from *Lasiodiplodia theobromae* Inhibits BAX-Triggered Cell Death and Suppresses Immunity Responses in *Nicotiana benthamiana*

**DOI:** 10.3390/plants11111462

**Published:** 2022-05-30

**Authors:** Qikai Xing, Yang Cao, Junbo Peng, Wei Zhang, Jiahong Wu, Yueyan Zhou, Xinghong Li, Jiye Yan

**Affiliations:** 1 Beijing Key Laboratory of Environment Friendly Management on Fruits Pests in North China, Institute of Plant Protection, Beijing Academy of Agriculture and Forestry Sciences, Beijing 100097, China; xingqikai@ipepbaafs.cn (Q.X.); caoyang@ipepbaafs.cn (Y.C.); pengjunbo@ipepbaafs.cn (J.P.); zhangwei@ipepbaafs.cn (W.Z.); zhouyueyan@ipepbaafs.cn (Y.Z.); lixinghong@ipepbaafs.cn (X.L.); 2 College of Plant Protection, Jilin Agricultural University, Changchun 130118, China; 20190110@mails.jlau.edu.cn

**Keywords:** secreted proteins, effector, *Lasiodiplodia theobromae*, plant immunity, susceptibility

## Abstract

*Lasiodiplodia theobromae* is a causal agent of grapevine trunk disease, and it poses a significant threat to the grape industry worldwide. Fungal effectors play an essential role in the interaction between plants and pathogens. However, few studies have been conducted to understand the functions of individual effectors in *L. theobromae*. In this study, we identified and characterized a candidate secreted effector protein, LtCSEP1, in *L. theobromae*. Gene expression analysis suggested that transcription of *LtCSEP1* in *L. theobromae* was induced at the early infection stages in the grapevine. Yeast secretion assay revealed that LtCSEP1 contains a functional signal peptide. Transient expression of *LtCSEP1* in *Nicotiana benthamiana* suppresses BAX-trigged cell death and significantly inhibits the flg22-induced PTI-associated gene expression. Furthermore, the ectopic expression of *LtCSEP1* in *N. benthamiana* enhanced disease susceptibility to *L. theobromae* by downregulating the defense-related genes. These results demonstrated that LtCSEP1 is a potential effector of *L. theobromae*, which contributes to suppressing the plant’s defenses.

## 1. Introduction

Grapevine (*Vitis vinifera* L.) is an extensively grown and economically important worldwide fruit crop [1]. Diseases caused by bacteria, fungi, oomycetes, and viruses have long been one of the important factors restricting the healthy development of the grapevine industry [2]. Botryosphaeria dieback caused by Botryosphaeriaceae species has become one of the most important grapevine trunk diseases [3,4,5,6]. Botryosphaeria dieback is caused by more than 20 Botryosphaeriaceae species, and the most common isolates are *Botryosphaeria dothidea*, *Diplodia mutila*, *Diplodia seriata*, *Lasiodiplodia theobromae*, and *Neofusicoccum parvum* [7,8,9]. *L. theobromae*, which can infect more than 500 fruit crops or woody trees, was reported to be one of the most aggressive woody plant-degrading pathogens in vineyards [10,11,12]. The fungi infect hosts via natural openings or pruning wounds, causing significant damage to plant production as a result of vascular discoloration, trunk canker, and eventual death of the wood [13,14,15]. Therefore, there is an urgent need for establishing effective and eco-friendly strategies to control the pathogen. However, a lack of understanding of the molecular mechanisms of *L. theobromae* restricts this goal.

During plant and pathogen interaction, plants have developed two layers of the immune system to recognize and to respond to pathogens: pathogen-associated molecular patterns (PAMPs)-triggered immunity (PTI) and effector-triggered immunity (ETI) [16,17,18]. Microbial effectors are usually small, cysteine-rich, secreted proteins that act in apoplast or specialized biotrophic interfaces, or that are translocated into plant cytoplasm [19,20,21,22]. The effectors can either contribute to pathogen virulence by undermining plant defense circuitry or be directly or indirectly recognized by corresponding resistance proteins (R proteins), leading to ETI [23,24]. The effectors function in a variety of ways: they can adjust the stomata behavior and reduce callose deposits; inactivate toxic compounds or enzymes produced by the plant host that are harmful to pathogens; subvert plant hormone signaling; or manipulate transcription of plant defense-related genes to promote the growth, development, and colonization of the pathogens [25,26,27]. Therefore, functional analysis of effector proteins is critical for elucidating the mechanisms of the colonization and the pathogenicity of the pathogens. 

In recent years, researchers have focused on pathogen isolation and identification, population structure, infection process and circulation, and secondary metabolites of *L. theobromae* [8,9,28]. Studies on fungal genetics and plant pathology have been significantly facilitated by genomic sequencing [29,30,31,32]. Whole-Genome sequencing and comparative transcriptome analyses of *L. theobromae* have revealed a large repertoire of over 500 *L. theobromae* candidate secreted effector proteins (LtCSEPs) [33,34]. In a large-scale screening, seven *L. theobromae* putative effectors were reported to suppress the *Burkholderia glumae*-induced hypersensitive cell death (HR) [33]. Besides, a set of *LtCSEP* genes was induced during the early infection of *L. theobromae* [34]. Taken together, *LtCSEP* genes are likely to play vital roles in plant immunity at the early infection stage. The secretory endopolygalacturonase protein LtEPG1 plays essential roles in *L. theobromae* virulence, and it acts as an elicitor [35]. Nevertheless, few *L. theobromae* effectors have been confirmed and characterized, and the function of individual effector is still unclear during infection.

It has been previously demonstrated that BR1.397, candidate secreted effector protein 1 (named hereafter as *LtCSEP1*; NCBI protein ID XP_035372397.1), can suppress the *B. glumae*-induced HR in *N. benthamiana* [33]. In this study, we demonstrate that the expression of *LtCSEP1* was abundantly induced during *L. theobromae* infection. Further, we found that transient expression of *LtCSEP1* could suppress BAX-triggered cell death and the induction of PTI-associated marker genes in *N. benthamiana*. *L. theobromae* can infect tobacco, so tobacco can be used as a host plant to study the infection mechanism of this pathogen [36]. Overexpressing *LtCSEP1* in *N. benthamiana* enhanced disease susceptibility to *L. theobromae* by downregulating defense-related genes. Collectively, this study indicates that *LtCSEP1* is an essential effector in *L. theobromae*, which contributes to manipulating the plant’s immunity.

## 2. Results

### 2.1. LtCSEP1 Is Transcriptionally Up-Regulated during Lasiodiplodia theobromae Infection

**Figure 1:**
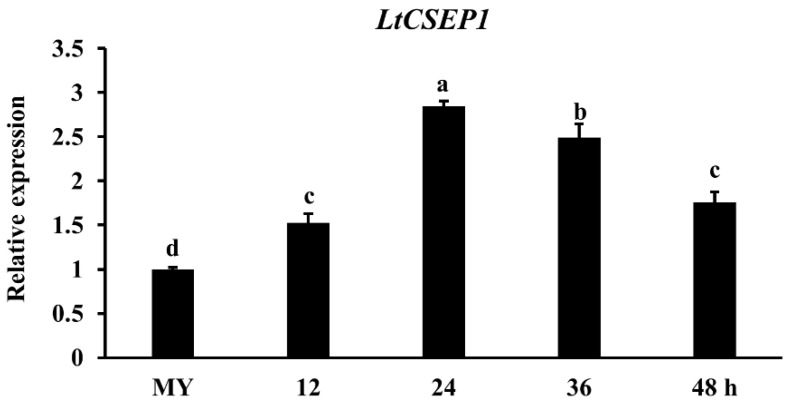
Relative expression levels of *LtCSEP1* during *Lasiodiplodia theobromae* infection. The expression of *LtCSEP1* was determined by real-time quantitative PCR (qRT-PCR) at 12, 24, 36, and 48 h post-*L. theobromae* inoculation (hpi). Total RNA was isolated from grapevine stems infected with *L. theobromae* strain CSS-01s. The relative expression level of *LtCSEP1* at various time points was compared with that in *L. theobromae* mycelium grown in a PDA broth, which was set to 1. The housekeeping gene *Actin* of *L. theobromae* was used as the internal standard to normalize the *LtCSEP1* expression in each sample. MY, *L. theobromae* mycelia grown in PDA plates for 48 h. The error bars indicate the mean values of three independent biological repetitions with standard errors. Different letters on top of the bars represent significant differences (*p* < 0.05) determined by Duncan’s multiple range test.

To determine the expression pattern of *LtCSEP1* during *L. theobromae* infection, the highly virulent isolate CSS-01s was inoculated into the stems of the grape cultivar summer black that is highly susceptible to CSS-01s [32]. The expression pattern of *LtCSEP1* in *L. theobromae* during different infection stages on wounded grapevine stems was investigated by qRT-PCR. The expression of *LtCSEP1* was significantly induced during the infection stages with a 3-fold increase at 24 hpi and a 2.5-fold increase at 36 hpi compared to its expression at the mycelial stage and then decreased gradually (Figure 1). These results indicate that *LtCSEP1* is induced in the early stages of infection of *L. theobromae* in the grapevine.

### 2.2. LtCSEP1 Encodes a Secretory Effector Protein

**Figure 2:**
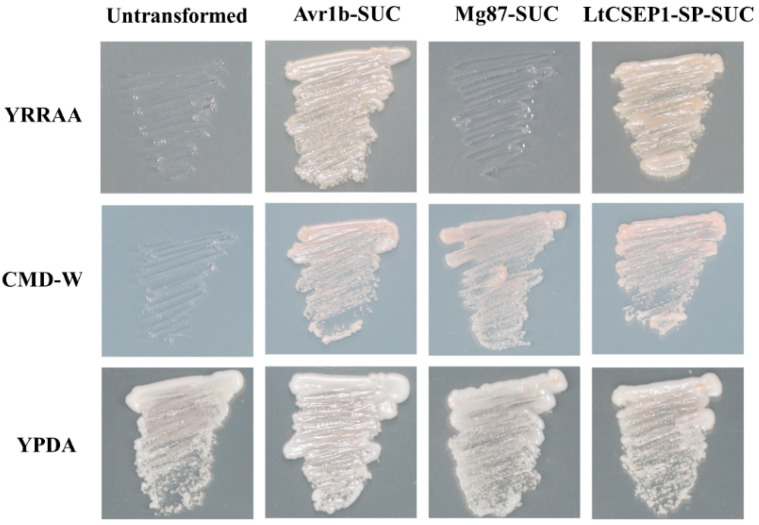
Functional validation of the putative signal peptide of LtCSEP1 using the yeast invertase secretion assay. The putative signal peptide sequence of *LtCSEP1* was fused in-frame to the invertase gene in the pSUC2 plasmid and transformed into yeast YTK12 strain. The signal peptide of secreted effector Avr1b from *Phytophthora sojae* and the first 25 amino acids of the non-secreted Mg87 protein from *Magnaporthe oryzae* were used as positive and negative controls, respectively.

Based on the sequence analysis, the putative effector LtCSEP1 contains 380 amino acids with a predicted molecular size of 41 kD (Figure S1). The 16 amino acids located at the N-terminal of LtCSEP1 were predicted to be the SP of LtCSEP1 (Figure S1). A yeast secretion trap assay based on the secretion of invertase for yeast growth on media with raffinose or sucrose as the sole carbon source has been developed to test the secretion function of the predicted SP [37,38]. Therefore, we performed the yeast secretion assay to validate the predicted secretory function of LtCSEP1. All the yeast strains grew on YPDA media while the yeast transformants carrying the pSUC2 grew on CMD-W (Figure 2). The invertase secretion deficient yeast strain YTK12 and the transformants with the negative control Mg87-SUC did not show any growth on the YPRAA medium. However, yeast transformants carrying the LtCSEP1-SP-SUC and positive control Avr1b-SUC rescued the growth of parental strain YTK12 on YPRAA medium, indicating that the SP of LtCSEP1 is functional. 

### 2.3. LtCSEP1 Suppresses BAX-Induced Cell Death in Nicotiana benthamiana

**Figure 3:**
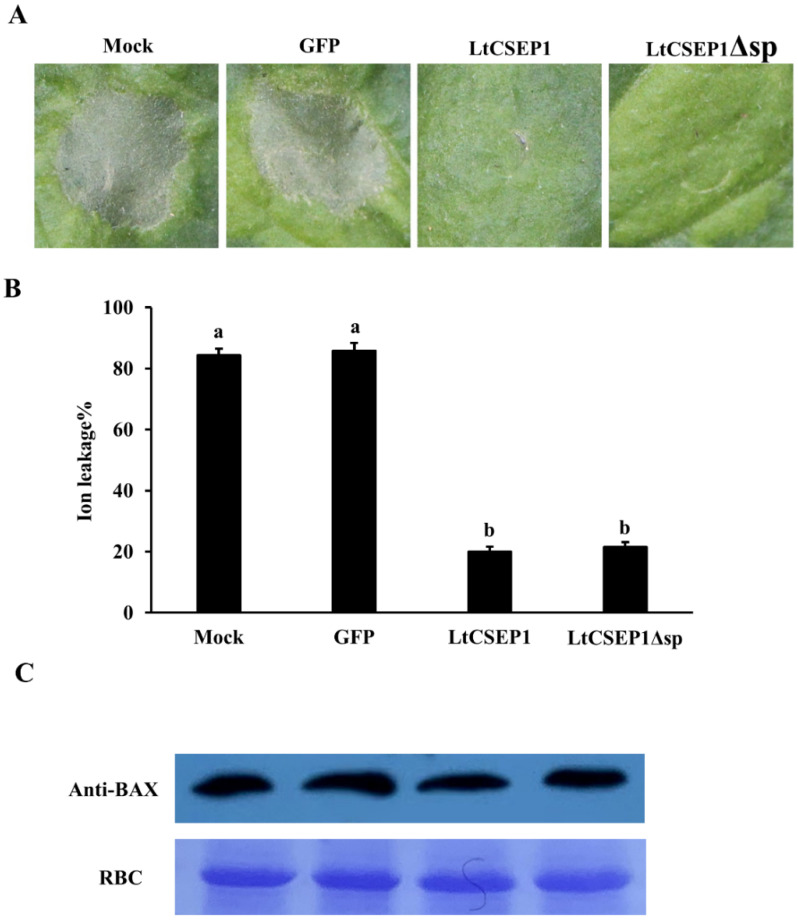
LtCSEP1 suppressed BAX-triggered cell death in *Nicotiana benthamiana*. (**A**) *N. benthamiana* leaves were infiltrated with *Agrobacterium tumefaciens* GV3101 containing pGR107 vector carrying Green fluorescent protein (GFP), LtCSEP1 or LtCSEP1Δsp (LtCSEP1 without the signal peptide sequence), respectively, followed by expression of the BAX 12 h later. Photos were taken 5 days post infiltration. The experiments were repeated three times with similar results. (**B**) Quantification of cell death by measuring ion leakage in the infiltrated *N. benthamiana* leaves. Ion leakage from leaf discs infiltrated with Agrobacterium cells was measured at 3 days post infiltration of BAX. Different letters represent significant differences (*p* < 0.05). (**C**) The presence of BAX proteins was detected by western blotting with anti-BAX antibodies.

The BAX-induced cell death symptoms in *N. benthamiana* resemble the HR exhibited by the plant’s immune system [39,40,41]. Therefore, the ability to suppress this BAX-induced cell death in *N. benthamiana* proved to be useful to identify the immunosuppressive effectors in *L. theobromae* [39,40,41]. To investigate whether *LtCSEP1* could suppress BAX-triggered cell death, *Agrobacterium* strains carrying *GFP*, *LtCSEP1*, and the *LtCSEP1* without the SP (*LtCSEP1Δsp*) were infiltrated into the *N. benthamiana* leaves. Twelve hours post first infiltration, the *Agrobacterium* strain with BAX were infiltrated in the same place, separately. The results showed that cell death triggered by BAX were almost totally suppressed on leaves co-expressing BAX and LtCSEP1 or LtCSEP1Δsp (Figure 3A). The expression of *GFP*, *BAX*, and *LtCSEP1* were confirmed by RT-PCR (Figure S2), and the accumulations of BAX proteins in leaves were also validated by western blotting (Figure 3C). Consistently, results showed that the ion leakage from *N. benthamiana* leaves co-expressing BAX and LtCSEP1 or LtCSEP1Δsp was significantly lower compared to the leaves expressing GFP (Figure 3B). These data demonstrated that LtCSEP1 has the ability to suppresses BAX-induced cell death, and it may play a role in limiting plant immunity. 

### 2.4. LtCSEP1 Suppresses flg22-Triggered PTI in N. benthamiana 

**Figure 4:**
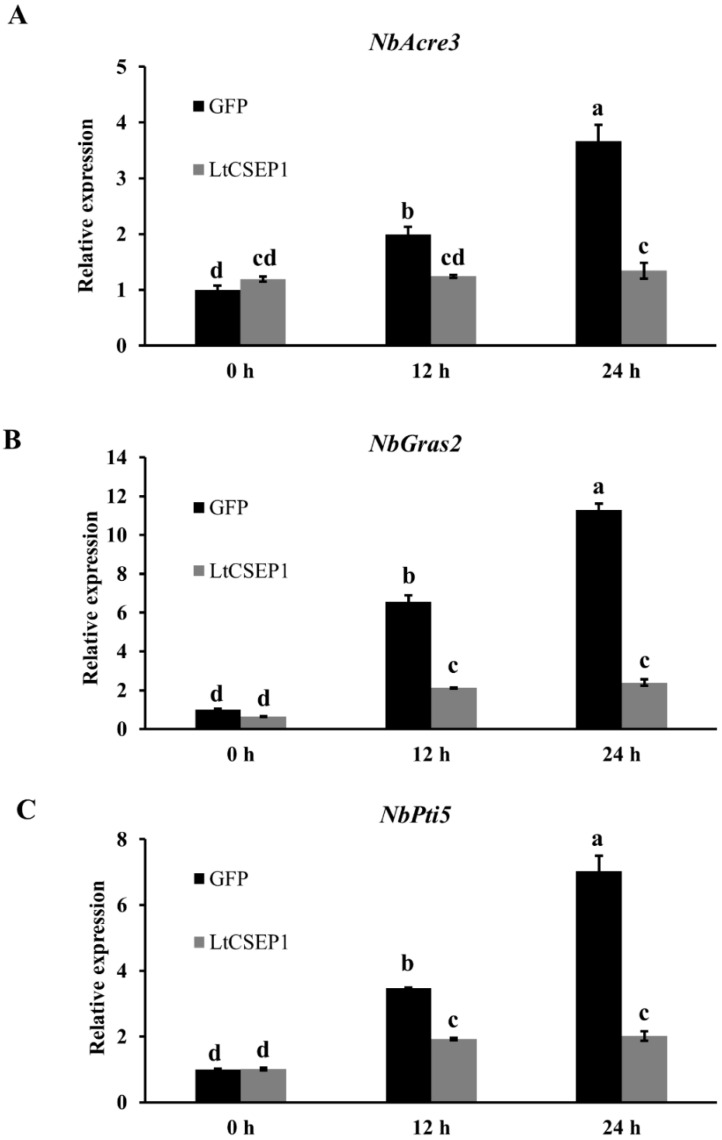
LtCSEP1 suppresses flg22-induced upregulation of PTI-associated genes in *Nicotiana benthamiana*. (**A**–**C**) The expression of three PTI-associated genes *NbAcre31* (**A**), *NbGras2*(**B**), and *NbPti5* (**C**) in *N. benthamiana* leaf tissues expressing LtCSEP1 or the negative control GFP after the treatment of flg22 was determined using qRT-PCR, respectively. The expression level of *NbEF1α* was used as an internal reference. Error bars indicate the mean values of three independent biological repetitions with standard errors. Different letters represent significant differences (*p <* 0.05).

To further investigate the effects of *LtCSEP1* expression on plant immunity, *LtCSEP1* and the control *GFP* were transiently expressed in *N. benthamiana* leaves and PTI-associated marker gene expression levels, including *Avr9/Cf-9 rapidly elicited 31*(*NbAcre31*), *GAI, RGA and SCR 2* (*NbGras2*), and *Pto-interacting 5* (*NbPti5*), were assessed by qRT-PCR after flg22 treatment. The results showed that expression of PTI-associated marker genes, *NbAcre31*, *NbGras2,* and *NbPti5*, was significantly induced by flg22 in the GFP control at 12 hpi and 24 hpi compared to their expression of untreated leaves at 0 hpi (Figure 4). In contrast, flg22 induced expression of PTI-associated genes was significantly suppressed in the leaves expressing the *LtCSEP1* (Figure 4). The expression of *GFP*, *BAX*, and *LtCSEP1* were confirmed by RT-PCR (Figure S3). To further elucidate whether LtCESP1 suppresses PAMP-triggered ROS generation, ROS production was examined in *N. benthamiana* leaves. The results showed that transient expression of LtCESP1 suppressed the flg22-induced ROS production, in contrast with the control GFP (Figure S4). These results indicate that LtCSEP1 suppressed the flg22-triggered PTI in *N. benthamiana*. 

### 2.5. LtCSEP1 Expression Enhances Plant Susceptibility to Lasiodiplodia theobromae

**Figure 5:**
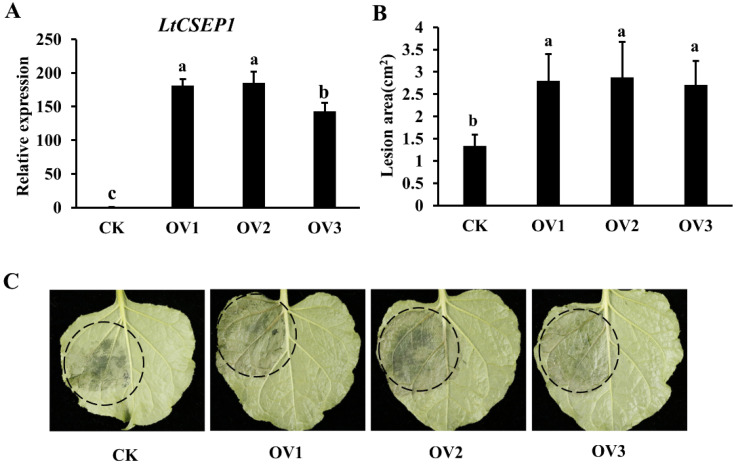
Overexpression of LtCSEP1 in *Nicotiana benthamiana* enhanced the susceptibility of plants to *Lasiodiplodia theobromae*. (**A**) Transcript levels of LtCSEP1 in transgenic overexpression lines (OV1, OV2, and OV3) as revealed by qRT-PCR. The expression of *NtEF1α* was used as the endogenous control. CK, the wild type *N. benthamiana* plants. (**B**) Lesion areas on *LtCSEP1* transgenic lines infected with *L. theobromae* CSS-01s at 5 days post-infection (dpi). At least ten leaves from each line were inoculated with *L. theobromae* CSS-01s. (**C**) Typical leaves with lesions resulting from *L. theobromae* 5 days post-infection (dpi). Error bars indicate the mean values of three independent biological repetitions with standard errors. Different letters represent significant differences (*p <* 0.05).

*N. benthamiana*, which is easily genetically transformed and has a short life cycle, is an ideal model plant to study the molecular mechanism of *L. theobromae* [36]. In order to further explore the biological function of *LtCSEP1*, we transformed Cam35S:LtCSEP1Δsp-GFP construct into *N. benthamiana*, and we obtained 20 independent *LtCSEP1* transgenic overexpressing *N. benthamiana* lines. Six independent homozygous *LtCSEP1*-transgenic lines were generated. Three *LtCSEP1* transgenic lines (OV1, OV2, and OV3) with the higher expression level of *LtCSEP1* (Figure 5A) were selected for further research. The results showed that the lesion areas in *LtCSEP1* transgenic overexpressing lines were obviously larger than those in wild-type control (Figure 5B,C).

**Figure 6:**
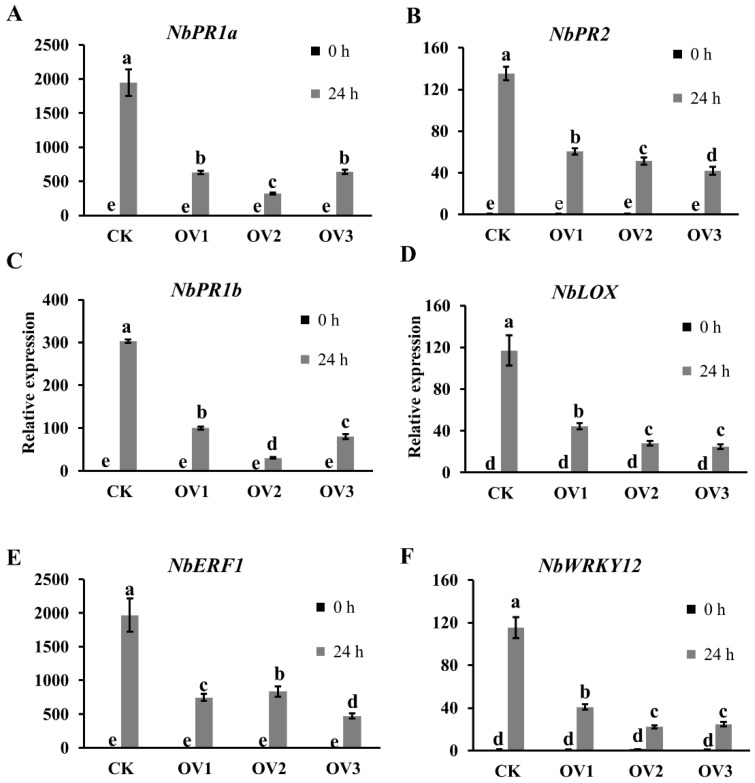
Downregulation of defense-related genes mediated by *LtCSEP1* in *LtCSEP1* transgenic overexpressing *N. benthamiana* lines. Transcript level of the *NbPR1b* (**A**), *NbPR2* (**B**), *NbPR1b* (**C**), *NbLOX* (**D**), *NbERF1* (**E**), and *NbWRKY12* (**F**) genes in LtCSEP1 transgenic lines during *L. theobromae* infection. Bars indicate means of three independent replicates and different letters indicate significant differences (*p* < 0.05).

To further elucidate the role of *LtCSEP1* in plant immune responses, the expression of defense-related genes in the SA, JA, and ET signaling pathways, including *pathogenesis-related protein 1A* (*NbPR1a*), *pathogenesis-related protein 2* (*NbPR2*)*, pathogenesis-related protein 1B* (*PR1b*), *linoleate 9S-lipoxygenase 5* (*NbLOX*), *ethylene-responsive transcription factor 1* (*NbERF1*), and *WRKY transcription factor12* (*NbWRKY12*), was significantly downregulated in *LtCSEP1* transgenic lines during *L. theobromae* infection compared to wild-type plants (Figure 6). These results suggested that the expression of LtCSEP1 inhibited plant immune responses in *N. benthamiana*.

## 3. Discussion

In the past decade, *L. theobromae* has emerged as a major pathogen infecting a wide range of crops worldwide. Pathogen effectors play essential roles in plant-pathogen interactions [19,20,21,22]. *L. theobromae* genome encodes more than 500 candidate effector secreted proteins [33,34]. However, little is known about the molecular mechanism of the individual effectors in *L. theobromae*. In this study, we demonstrated that *LtCSEP1* in *L. theobromae* acts as an effector that suppresses plant immunity in *N. benthamiana*.

The presence of an N-terminal signal peptide, is one of the hallmarks of secretory proteins from fungal pathogens, which was exported by the ER-Golgi secretion pathway [42]. LtCSEP1 was predicted to contain the SP at the first 16 amino acid of the N-terminus (Figure S1). In our study, the prediction was supported using the yeast secretion assays, in which the SP of LtCSEP1 was functional to guide the secretion of the truncated invertase out of yeast cells (Figure 2). A common feature of functional effectors is that genes are normally transcriptionally regulated when a pathogen infects the plant host [43]. Our result revealed that the expression of *LtCSEP1* was significantly up-regulated at the early infection stage of *L. theobromae* (Figure 1). It is well known that the effectors from plant pathogens are capable of suppressing immunity-associated cell death induced by elicitors and thus promoting the invasion, colonization, and expansion of the pathogens [44,45]. For example, most of the candidate effectors in *Botryosphaeria dothidea* which can infect hundreds of woody plants were reported to suppress BAX-triggered cell death in *N. benthamiana* [46]. Besides, 7 of 70 candidate effectors that were identified in *Valsa mali*, which is aggressive to many Rosaceae woody plants, were able to suppress BAX-triggered cell death in *N. benthamiana* [47]. Here, LtCSEP1 was also shown to suppress BAX-induced cell death when transiently expressed in *N. benthamiana* leaves. Furthermore, we demonstrated that the overexpression of *LtCSEP1* significantly suppressed three PTI-associated genes in *N. benthamiana*. These results demonstrated that LtCSEP1 acts as a suppressor of plant immunity.

Previous studies reported that the heterologous expression of pathogen effectors in model plants is an important gain-of-function strategy for studying the function of individual effector [40]. It has been reported that ectopic expression of the MIF-like effector MiMIFs in *Arabidopsis* significantly suppressed plant immune responses and increased the infectiousness of *Meloidogyne incognita* [48]. It was indicated that PsCRN115- and PsCRN161-transgenic lines in *N. benthamiana* significantly improved disease resistance to oomycete pathogens and exhibited enhanced tolerance to abiotic stresses [49,50]. Due to the lack of a stable gene knock-out and knock-down system for *L. theobromae* at present, ectopic expression of individual effector in *N. benthamiana* can be used to investigate the molecular mechanism of the pathogen [36]. In this study, we found that the overexpression of *LtCSEP1* enhanced plant susceptibility to *L. theobromae* infection (Figure 5). In response to pathogens, host plants employ multiple layers of immune responses, which were orchestrated by a complex network of the phytohormone signaling pathway [51]. Plant pathogens have evolved diverse strategies to subvert phytohormone signaling networks that interfere with host immunity, thus leading to the susceptibility of host plants [52]. *NbPR1a* and *NbPR2* are marker genes for salicylic acid (SA)-dependent defense [53]. *NbLOX* and *NbPR1b* are marker genes for jasmonic acid (JA)-dependent defense [54]. The *NbERF1* is one marker gene for the ethylene-mediated signaling pathway [55]. Gene expression analysis showed that these defense-related genes were downregulated in *LtCSEP1* transgenic plants compared to wild-type plants post *L. theobromae* inoculation (Figure 6). These results indicated that LtCSEP1 may inhibit plant immune responses by regulating a series of defence-related genes.

Pathogen effectors were reported to be delivered into specific host cell compartments, and they physically interacted with host proteins [19,20,21,22]. Host proteins targeted by the *LtCSEP1* effector in grapevines remain to be further screened and identified through yeast two-hybrid (Y2H) or co-immunoprecipitation (Co-IP) assays, which can help to further investigate how LtCSEP1 manipulates host immunity [19,20,21,22]. Overall, a novel secreted protein *LtCSEP1* in *L. theobromae* was identified and characterized as an effector which suppresses plant immunity in *N. benthamiana*. However, the precise molecular function of LtCSEP1 in the interaction between grape and *L. theobromae* remains to be further elucidated.

## 4. Materials and Methods

### 4.1. Fungal and Bacterial Isolates, Plant Materials, and Growth Conditions

The virulent *L. theobromae* strain CSS-01s used in this study was deposited at the Beijing Academy of Agricultural and Forestry Sciences, Beijing, China and cultured in Potato dextrose agar medium (PDA: 200 g fresh potato, 20 g dextrose and 20 g agar per liter). Green shoots of *Vitis vinifera* var. summer black which is susceptible to the *L. theobromae* were grown and maintained in a controlled growth house at 26 °C for three months at the Beijing Academy of Agriculture and Forestry Sciences, Beijing, China. *N. benthamiana* plants were grown and maintained in a controlled growth chamber under a 16 h light/8 h dark cycle at approximately 26 °C. *Agrobacterium tumefaciens* GV3101 and EHA105 were cultured in Luria-Bertani broth (LB: 5 g yeast extract, 10 g tryptone, 10 g NaCl per liter) supplemented with their respective antibiotics at 28 °C in a shaking incubator at 180 rpm. The concentrations of antibiotics used in this study are: kanamycin, 50 μg/mL and rifampin, 25 μg/mL. Yeast stain YTK12 was cultured in YPDA liquid medium (10 g yeast extract, 20 g peptone, 20 g glucose, 0.03 g adenine hemisulphate, and 20 g agar per liter) at 30 °C. All experiments in this study have been repeated independently at least three times with similar results.

### 4.2. Lasiodiplodia theobromae Inoculation

Stem inoculations were conducted as previously described with minor modifications [33]. The *L. theobromae* isolate CSS-01s was cultured on a PDA medium at 26 °C for three weeks to generate conidia. The conidia were eluted with sterile water containing 0.02% Silwet L-77. Green stems of *Vitis vinifera* summer black were wounded using a 4-mm corkborer, then inoculated with *L. theobromae* conidial suspension (1 × 10^6^ conidia mL^−1^), and maintained in an inoculation room at 26 °C with a relative humidity of 90%. Then, shoot phloem within a 2.0- to 3.0-cm range from the wound point from at least ten infected stems were collected at 12, 24, 36, and 48 h post-inoculation (hpi). Simultaneously, fungal mycelia were collected from PDA plates at 48 h post-culturing. All the collected samples were immediately frozen in liquid nitrogen and then stored at −80 °C.

### 4.3. RNA Isolation and Quantitative Real-Time RT-PCR

Total RNA from 100 mg *L. theobromae* hyphae or plant tissues was isolated with Trizol Reagent (Invitrogen, Carlsbad, CA, USA) according to the manufacturer’s instructions. The RNA concentration was quantified using a NanoDrop 2000c spectrophotometer (Thermo Scientific, Waltham, MA, USA) and a total of 3 μg of isolated RNA was treated with Ambion™ DNase I (Invitrogen) and then reverse transcribed to cDNA using the SuperscriptTM III First-Strand Synthesis SuperMix kit (Invitrogen). Quantitative real-time PCR (qRT-PCR) was performed in a quantitative PCR thermal cycler using a 7500 real-time PCR system (Applied Biosystems, Foster City, CA, USA) with TB Green^®^ Premix Ex Taq™ II (Tli RNaseH Plus) (TaKaRa, Dalian, China). Each reaction was prepared in a 15 μL final volume using 1.0 μL cDNA, 0.5 μL respective primer (10 μM), 0.3 μL ROX reference dye, 5.2 μL of sterile water, and 7.5 μL 2 × TB Green Premix Ex Taq II (Tli RNaseH Plus). The amplification conditions were as follows: 2 min for denaturation at 95 °C, followed by 40 cycles of 95 °C for 5 s and 60 °C for 35 s. For the analysis of relative expression during infection, the *Actin* gene of *L. theobromae* was used as the internal reference, whereas, for other analyses, the *NtEF1α* gene of *N. benthamiana* was used. Relative gene expression was calculated using the 2^−ΔΔCt^ method [56]. The experiment was conducted using three independent replicates for each timepoint. All primers are listed in Table S1.

### 4.4. Cloning of the LtCSEP1 cDNA Sequence

The coding sequence (CDS) of the *LtCSEP1* gene based on the *L. theobromae* sequence (NCBI Accession XM_035512929.1) was PCR-amplified from the cDNA of *L. theobromae*. The PCR product was subcloned into the pMD18-T vector (Takara), transformed into *Escherichia coli* DH5α-competent cells and verified by sequencing. The signal peptide of LtCSEP1 was predicted by the SignalP 4.1 server with the default settings (http://www.cbs.dtu.dk/services/SignalP/ (accessed on 6 January 2019)).

### 4.5. Functional Validation of the Predicted Signal Peptide Using Yeast Secretion Assay

Functional validation of the predicted signal peptide (SP) of LtCSEP1 was performed using a yeast secretion trap assay [37]. The plasmid pSUC2 carries a truncated invertase gene, SUC2, without the initial methionine and signal peptide [41]. The predicted signal peptide sequence of LtCSEP1, a 0.12-kb fragment from the start codon of LtCSEP1, was cloned from the cDNA of *L. theobromae* and subcloned into the EcoR I and the Xho I sites of the plasmid pSUC2. The fusion plasmids were then transformed into the invertase secretion-defective yeast strain YTK12 using the Frozen-EZ yeast transformation II kit (Zymo Research, Irvine, CA, USA). Transformants were plated on YPDA medium, CMD-W medium (6.7 g yeast nitrogen base without amino acids, 0.7 g tryptophan dropout supplement, 20 g sucrose, 1 g glucose, and 20 g agar per liter), and YPRAA medium (10 g yeast extract, 20 g peptone, 20 g raffinose, 2 mg antimycin A per liter) [37]. The predicted SP of Avr1b from *Phytophthora sojae* and the first 25 amino acids of the non-secreted Mg87 protein from *Magnaporthe oryzae* were used as a positive and a negative control, respectively. The functionality of the predicted SP was determined by colony growth on the YPRAA medium. Plates were photographed five days post-incubation at 30 °C.

### 4.6. Cell-Death Suppression Assay in Nicotiana Benthamiana

The CDS of *LtCSEP1* and its truncated variant lacking the SP were amplified and subcloned into plasmid pGR107 [41] using an In-Fusion HD Cloning Kit (Clontech, Mountain View, CA, USA). All constructs were verified by sequencing and transformed into *A. tumefaciens* strain GV3101. The *A. tumefaciens* strains containing the corresponding plasmid were infiltrated into four-weeks-old *N. benthamiana* leaves with needleless syringes, followed by the infiltration of the *A. tumefaciens* carrying BAX 12 h post the initial infiltration. Four *N. benthamiana* leaves from two seedlings were used for each combination. Cell death symptoms were photographed 5 days post the final infiltration. The BAX-triggered cell death in *N. benthamiana* was quantified based on ion leakage in the agroinfiltrated leaves as previously described [30]. Ion leakage was measured three days after infiltration of BAX. At least ten leaf discs (5 mm diameter) were collected and the experiment was repeated three times. 

### 4.7. PTI-Associated Gene Expression and ROS Assays in Nicotiana benthamiana

The experiment was performed as previously reported [33]. Briefly, leaves from four *N. benthamiana* plants infiltrated by *A. tumefaciens* cells carrying *LtCSEP1* or the GFP were sprayed with 1 µM flg22 (Sangon Biotech, Shanghai, China) 12 h post the initial infiltration. The expression of three PTI-associated genes *NbAcre31*, *NbGras2*, and *NbPti5* was evaluated by qRT-PCR. Four *Nicotiana benthamiana* leaves were collected 12 and 24 h after infiltration of flg22. For chemiluminescence detection of ROS burst, leaf disks (5 mm in diameter) were collected and suspended into a 96-well plate containing double-distilled water overnight. Then, the water was withdrawn and replaced with 100 mL of luminol solution containing 200 mM luminol (Sigma), 10 mg/mL horseradish peroxidase (Sigma). Chemiluminescence was continuously measured using a microplate reader (Tecan, Männedorf, Switzerland).

### 4.8. Nicotiana benthamiana Transformation and Pathogenicity Assays for L. theobromae

The vector Cam35S:LtCSEP1Δsp-GFP was transformed into *A. tumefaciens* strains EHA105. The *N.* *benthamiana* transformation was performed using the leaf disc transformation method as previously described [57]. The screening of the positively transformed plants was performed on an MS medium containing 30 μg/L of hygromycin. After rooting and acclimatization, the regenerated plants were grown in a greenhouse to set seeds by self-pollination, and transgenic T3 lines were used for the subsequent experiments. Four-week-old transgenic *N*. *benthamiana* lines and the wild-type plants were selected for the pathogenicity assay. The abaxial surfaces of ten detached *N*. *benthamiana* leaves were inoculated with *L. theobromae* conidial solution at a concentration of 1 × 10^6^ conidia mL^−1^, and they were maintained in an inoculation room at 26 °C with 90% humidity. At least ten leaves from each line were challenged with *L. theobromae* CSS-01s. The leaves were photographed five days post-inoculation and the lesion areas were measured using ImageJ. 

### 4.9. Protein Extraction and Western Blot

Total proteins of *N. benthamiana* leaves were extracted using a plant protein extraction kit (CWBIO, Beijing, China). The extracted proteins were separated by a 12% SDS-PAGE gel, then transferred to a PVDF membrane (Millipore, Billerica, MA, USA). A primary anti-GFP Bax monoclonal antibody (Abcam, Cambridge, UK) and an HRP-conjugated anti-mouse secondary antibody diluted 1:5000 (CWBIO) were used.

## Data Availability

The data presented in this study are available on request from the corresponding author.

## Supplementary Materials

The following supporting information can be downloaded at: https://www.mdpi.com/article/10.3390/plants11111462/s1, Figure S1: LtCSEP1 is predicted to be a secreted protein. Figure S2: Gene expression in *N. benthamiana* leaf tissues expressing GFP, LtCSEP1, and BAX was analyzed by reverse-transcriptase polymerase chain reaction (RT-PCR). Figure S3: Gene expression in *N. benthamiana* leaf tissues expressing LtCSEP1 or the control GFP after the treatment of flg22 was analyzed by RT-PCR. Figure S4: LtCSEP1 suppressed Flg22-induced ROS in *Nicotiana benthamiana*. Table S1: Primers sequences used in this study.
