# Supplementary material for: A Putative Effector LtCSEP1 from Lasiodiplodia theobromae Inhibits BAX-Triggered Cell Death and Suppresses Immunity Responses in Nicotiana benthamiana
Source: Plants (Basel). 2022 May 30;11(11):1462. doi: 10.3390/plants11111462 (PMC9182993; doi:10.3390/plants11111462)
Supplement: Supplementary file 1 [file plants-11-01462-s001.zip › plants-1676679-supplementary.pdf]

MRAAALIPLAIGLVAAGPVEKRQNFNFAAIAADRADEIAAISADNLGPVDPVV

LATTVVEATSAYDATAAIAAATSAVTAVVEKREACQTYSGAGPVVTSAPSDWV

NAEVLTNPALTADVPSGYEAAPSFTNLQGAVQQMGYLTVKTLDSYSPAQCASY

CDDEPLCMGFNVYFERDPSEDTSCASDGNPDSITTIACTLYAYHVAASKATNTG

QYRDNFQVVITGSNGYNKDSSKQSFTSIDGYQAPQNFEDACINAPTYNDFDSY

ITYTTYTDAYDPRVCAKACDAQTEYDKEHPNDDQEYKACNYFVAYVMAKNE

EPQGLFCALYSLPWNSTYAVNTGYSWSSDVYTIYNSLAYTVSGDLDFGNNEEI

EDFVYEDAN

Figure S1. LtCSEP1 is predicted to be a secreted protein. LtCSEP1 contains 380 amino acids with a predicted molecular size of 41 kD. The 16 amino acids located at the N-terminal of LtCSEP1 were predicted to be the signal peptide of LtCSEP1.

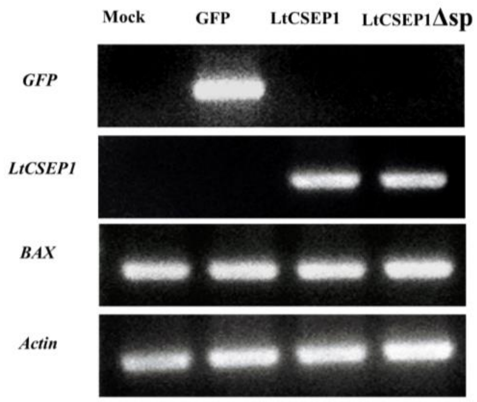

Figure S2. Gene expression in *N. benthamiana* leaf tissues expressing GFP, LtCSEP1 and BAX was analyzed by reverse-transcriptase polymerase chain reaction (RT-PCR). The actin gene of *N. benthamiana* was used as an internal standard.

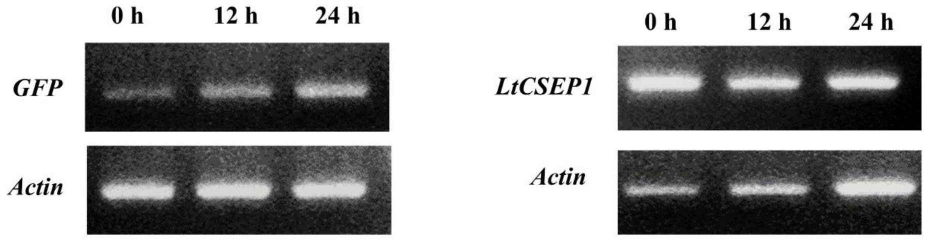

Figure S3. Gene expression in *N. benthamiana* leaf tissues expressing LtCSEP1 or the control GFP after the treatment of flg22 was analyzed by RT-PCR. The actin gene of *N. benthamiana* was used as an internal standard.

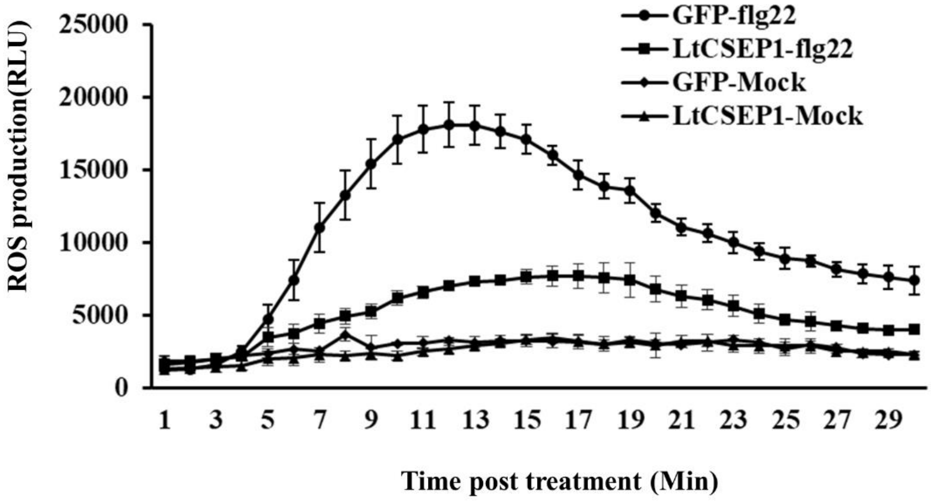

Figure S4. LtCSEP1 suppressed Flg22-induced ROS in *Nicotiana benthamiana*.

Table S1. Primers used in this study.

Primer name	Primer sequence (5'-3')
**Primers for vector construction**	
CSEP1SP-EF	TTTATGAATTCATGCGCGCCGCAGCCCTC
CSEP1SP-XR	TAATACTCGAGACGGTCAGCCGCAATAG
CSEP1B-SmF	ACCCGGGATGGGCCCTGTGGAAAAGC
CSEP1B-XIR	TCTCGACGTTGGCGTCTTCGTAAACG
CSEP1G-BIF	GGGATCCATGGGCCCTGTGGAAAAGC
CSEP1G-XIR	TCTCGACGTTGGCGTCTTCGTAAACG
**Primers for vector construction**	
Actin-RT-F1	AATCGTGAGGGATGTGAAGG
Actin-RT-R1	GCATTTTCTGTGCACAATGG
BAX-RT-F	ATCGATATGGACGGGTCCGGGGAG
BAXRT-R	GTCGACTCAGCCCATCTTCTTCCAGATGG
GFP-RT-F1	GACGTAAACGGCCACAAGTT
GFP-RT-F2	CTCCAGCAGGACCATGTGAT
CSEP1-RT-F	TACCTGACTGTCAAGACC
CSEP1-RT-R	TTGTAGATGGTGTAGACG
**Primers for qRT-PCR**	
NbEF1α-qF	AAGGTCCAGTATGCCTGGGTGCTTGAC
NbEF1α-qR	AAGAATTCACAGGGACAGTTCCAATACCA
LtActin-qF	TCTTCGCTCGAGAAGTCGTA
LtActin-qR	ACAATGGAAGGTCCGCTCTC
NbPR1a-qF	GTGCCCAAAATTCTCAACAAG
NbPR1a-qR	TTCTACACCTACATCTGCACGAG
NbPR2-qF	TCCAGATACAAATGTCTTCAACG
NbPR2-qR	TGGGACGTCGAGAATGATCT
NbPR1b-qF	GTGGACACTATACTCAGGTG
NbPR1b-qR	TCCAACTTGGAATCAAAGGG

NbLOX-qF	AGAAATGGATGTCCACCTTGA
NbLOX-qR	GGACTCATCCCAGTCAAATGTC
NbERF1-qF	GCGAAGTAAAATCGGAGCA
NbERF1-qR	CCTTCCCCTAGGTAACTCAGC
NbWRKY12-F	CTCATCAGCTAGTTCATTTGATGC
NbWRKY12-R	AGCTCGGTCTTTGTTCTAAAAGC
NbAcre31-qF	AATTCGGCCATCGTGATCTTGGTC
NbAcre31-qR	GAGAAACTGGGATTGCCTGAAGGA
NbGras2-qF	TACCTAGCACCAAGCAGATGCAGA
NbGras2-qR	TCATGAGGCGTTACTCGGAGCATT
NbPti5-qF	CCTCCAAGTTTGAGCTCGGATAGT
NbPti5-qR	CCAAGAAATTCTCCATGCACTCTGTC
